# Intraspecific N and P stoichiometry of *Phragmites australis*: geographic patterns and variation among climatic regions

**DOI:** 10.1038/srep43018

**Published:** 2017-02-24

**Authors:** Yu-Kun Hu, Ya-Lin Zhang, Guo-Fang Liu, Xu Pan, Xuejun Yang, Wen-Bing Li, Wen-Hong Dai, Shuang-Li Tang, Tao Xiao, Ling-Yun Chen, Wei Xiong, Yao-Bin Song, Ming Dong

**Affiliations:** 1 Key Laboratory of Hangzhou City for Ecosystem Protection and Restoration, College of Life and Environmental Sciences, Hangzhou Normal University, Hangzhou, China; 2 State Key Laboratory of Vegetation and Environmental Change, Institute of Botany, Chinese Academy of Sciences, Beijing, China; 3 Institute of Wetland Research, Chinese Academy of Forestry, Beijing, China

## Abstract

Geographic patterns in leaf stoichiometry reflect plant adaptations to environments. Leaf stoichiometry variations along environmental gradients have been extensively studied among terrestrial plants, but little has been known about intraspecific leaf stoichiometry, especially for wetland plants. Here we analyzed the dataset of leaf N and P of a cosmopolitan wetland species, *Phragmites australis*, and environmental (geographic, climate and soil) variables from literature and field investigation in natural wetlands distributed in three climatic regions (subtropical, temperate and highland) across China. We found no clear geographic patterns in leaf nutrients of *P. australis* across China, except for leaf N:P ratio increasing with altitude. Leaf N and N:P decreased with mean annual temperature (MAT), and leaf N and P were closely related to soil pH, C:N ratio and available P. Redundancy analysis showed that climate and soil variables explained 62.1% of total variation in leaf N, P and N:P. Furthermore, leaf N in temperate region and leaf P in subtropical region increased with soil available P, while leaf N:P in subtropical region decreased with soil pH. These patterns in *P. australis* different from terrestrial plants might imply that changes in climate and soil properties can exert divergent effects on wetland and terrestrial ecosystems.

Nitrogen (N) and phosphorus (P), two of the most abundant macroelements in plants, can control plant growth, alter species composition and influence ecosystem functioning^1^,^2^. Because of their significance, N and P in plants, especially in leaves, have been frequently studied in biogeochemistry, community ecology and ecosystem ecology^1^,^2^,^3^. For example, N:P ratio were used to infer the nutrient limitation of plant populations and communities^4^,^5^. Plant N and P have also been frequently investigated to explore the effects of environmental changes on biogeochemical cycling^6^,^7^. Besides, N and P of organisms are effective tools to study the nutrient and energy flows in food webs across multiple trophic levels^3^,^8^,^9^.

Studying variation in N and P of plants along environmental gradients can improve our understanding and prediction of the responses of plant tissue nutrients to environmental changes^2^,^10^. Previous interspecific studies have found that leaf N and P varied along geographic gradients, e.g., leaf N and P increased with latitude and altitude^11^,^12^,^13^,^14^. Several hypotheses, related to climate and soil, were proposed to explain these geographic patterns. First, the Temperature-Dependent Physiology Hypothesis^11^,^15^ predicts that tissue P increased more rapidly than N at lower temperature, and tissue N:P ratio increased with increasing temperature and decreasing latitude. The reason is that plants need more P-rich ribosomes than N-rich proteins to sustain growth at lower temperature^11^. Second, the Growing Season Duration Hypothesis^3^,^16^ predicts that plants at sites with shorter growing season (e.g. higher latitude) tend to grow more rapidly to achieve their life history, thus, they always have higher P, and lower N:P ratio. Third, the Environmental Nutrient Supply Hypothesis^17^,^18^,^19^ predicts that plant nutrient contents are strongly correlated with nutrient availability in the soil. The shift from N to P limitation in soils toward lower latitude makes tissue N:P ratio decreased with increasing latitude^17^,^18^,^19^.

However, most of previous studies on geographic variation in leaf N and P mainly focused on terrestrial plants and little on wetland plants. No clear evidence showed that whether there is the same latitudinal gradient in nutrient limitation in wetlands as that in terrestrial lands. Azonal distribution of wetland plants makes them showing weaker relationships with climate than terrestrial plants^20^. These two aspects might cause the geographic trends in leaf N and P of wetland plants to be different from that of terrestrial plants. To the best of our knowledge, intraspecific variation in leaf N and P of wetland plants were seldom investigated (but see Zhou *et al*.^21^). Investigating the geographic variation in leaf N and P within species can help to uncover the mechanism of relationships between plant tissue nutrients and environments^21^. In addition, it can exclude the confounding effects of taxonomic and phylogenetic structure like those found to influence the geographic patterns in leaf nutrients, and their linkages to climate and soil^22^,^23^,^24^. Although several previous works focused on the variation of plant tissue nutrients within species, most of them were conducted along relatively narrow environmental gradients^10^,^25^,^26^. To more accurately predict the responses of a single plant species to climate change, especially in terms of the leaf stoichiometry, large-scale studies are needed to fully determine the geographical pattern of leaf N and P at intraspecific level.

In this study, we aimed to explore the intraspecific patterns in leaf N and P of wetland species in relation to climate and soil variables in three climatic regions (subtropical, temperate and highland). Considering the differences between wetland and terrestrial ecosystems, we first hypothesized that there are geographic patterns in leaf N, P and N:P ratio of a wetland species, and they are different from what has been previously reported in terrestrial plants. Because wetlands are azonal and they are more influenced by local environmental factors^20^, we then hypothesized that leaf N, P and N:P ratio of a wetland species are less influenced by climate than by soil. At last, different climatic regions cover different parts of environmental gradients, thus, we hypothesized that leaf N, P and N:P ratio of a wetland species at different climatic regions are affected by different environmental factors.

To test these hypotheses, we used the data set on leaf N and P of the wetland plant *Phragmites australis* and environmental variables from published studies and our field investigation in natural wetlands across the species range in China. *Phragmites australis*, a cosmopolitan grass, is dominant in many wetland ecosystems. It distributed widely in different climatic regions from tropical to temperate regions in China as well as in the world^27^. Owing to both phenotypic plasticity and genetic variability, the variation in morphological and chemical traits of *P. australis* is considerable^27^,^28^,^29^. These characteristics made *P. australis* a suitable plant for studying the intraspecific variation in leaf N and P.

## Results

### Leaf N, P and N:P ratio of *P. australis* across China

**Figure 1:**
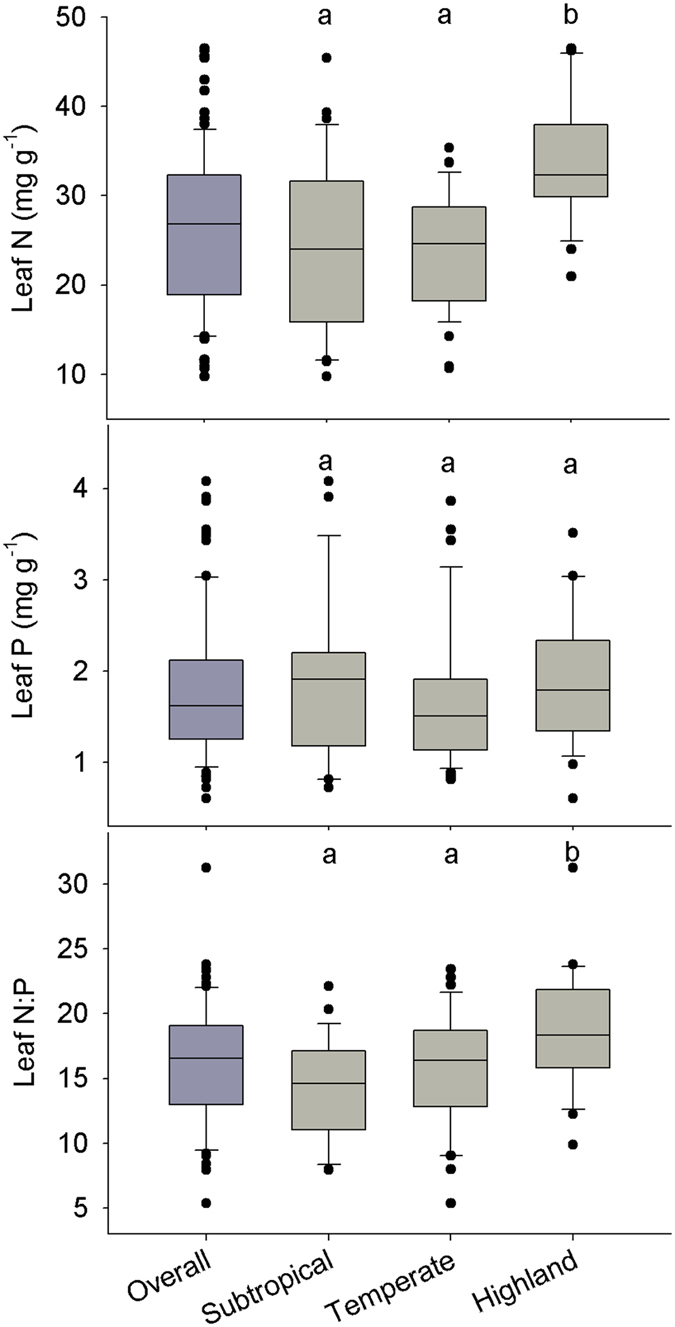
Leaf N, P and N:P ratio of *Phragmites australis* overall and in three climatic regions in China. Different letters indicate significant differences between climatic regions (*p* < 0.05; Tukey’s HSD).

The means (±SD) of leaf N, P and N:P ratio of *P. australis* were 26.4 ± 8.6 mg g^−1^, 1.8 ± 0.8 mg g^−1^ and 16.1 ± 4.6, respectively. Leaf P varied the most, ranging from 0.6 to 4.1 mg g^−1^, while leaf N:P ratio the least, ranging from 5.4 to 31.2, across the geographic range of *P. australis* in China (Fig. 1). Leaf N varied from 9.8 to 46.5 mg g^−1^ (Fig. 1).

One-way ANOVA showed that climatic regions had significant effects on leaf N (*F*_(2,92)_ = 12.85, *p* < 0.001) and N:P ratio (*F*_(2,80)_ = 6.34, *p* = 0.003), but not on leaf P (*F*_(2,85)_ = 0.74, *p* = 0.482). Leaf N in highlands (mean = 33.5, SD = 6.9) was significantly higher than that in subtropical (mean = 24.5, SD = 9.4) and temperate regions (mean = 23.9, SD = 6.5). Leaf N:P ratio in highlands (mean = 18.7, SD = 4.5) was significantly higher than in subtropical (mean = 14.3, SD = 1.9) and temperate regions (mean = 15.8, SD = 4.4).

### Effects of environmental variables on leaf N, P and N:P ratio of *P. australis* across China

**Figure 2:**
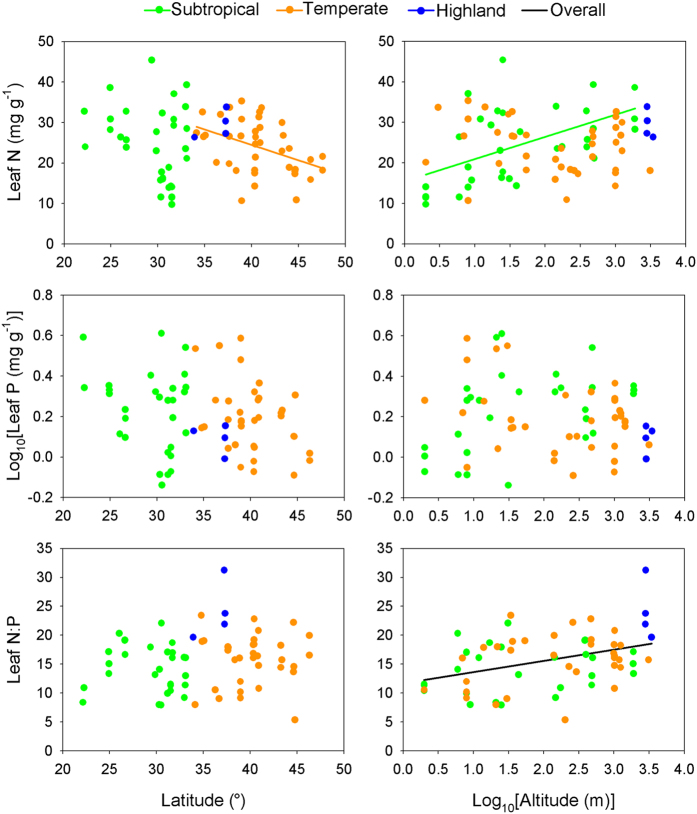
Relationships between leaf nutrients of *Phragmites australis* and geographic variables in three climatic regions. Lines are plotted for the relationships with *p* < 0.05.

**Figure 3:**
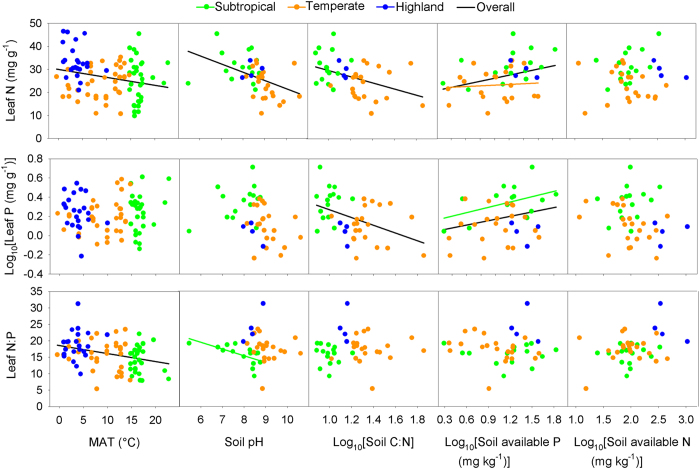
Relationships between leaf nutrients of *Phragmites australis*, climate and soil variables in three climatic regions. Lines are plotted for the relationships with *p* < 0.05. MAT stands for mean annual temperature.

Leaf N:P ratio of *P. australis* increased significantly with altitude (*t* = 2.48, *p* = 0.013), while leaf N and P didn’t show significant trends along latitudinal or altitudinal gradients (*p* > 0.05; Fig. 2). Leaf N of *P. australis* decreased with MAT (mean annual temperature; *t* = −2.59, *p* = 0.009), soil pH (*t* = −2.27, *p* = 0.023) and soil C:N ratio (*t* = −2.00, *p* = 0.046), but increased with soil available P (*t* = 2.79, *p* = 0.005; Fig. 3). Leaf P was negatively correlated with soil C:N ratio (*t* = −2.44, *p* = 0.015) and positively with soil available P (*t* = 2.26, *p* = 0.024; Fig. 3). Leaf N:P ratio decreased significantly with MAT(*t* = −2.69, *p* = 0.007; Fig. 3). Leaf nutrients are more correlated with soil variables than climate factors based on the number of significant relationships and R^2^ values (variance explained) (Fig. 3; Supplementary Table S1).

**Figure 4:**
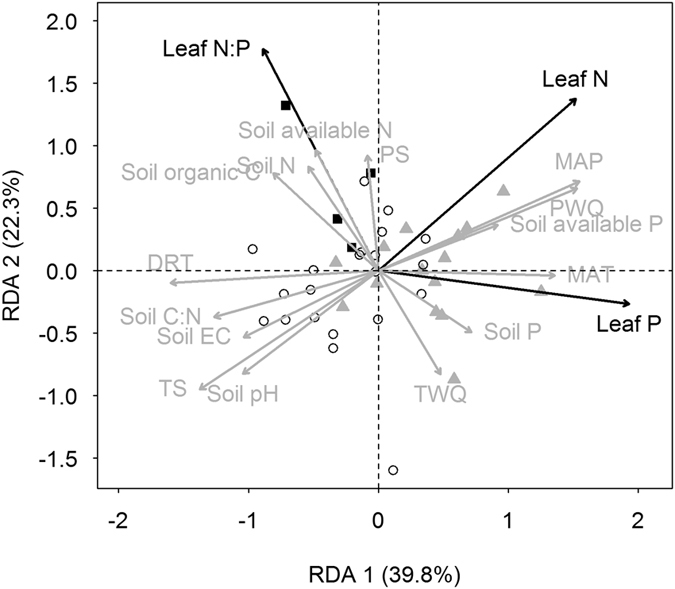
Redundancy analysis for the covariation among leaf nutrients (leaf N, P and N:P), soil and climate variables. Different symbols represent different climatic regions: 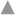, subtropical; ○, temperate; ■, highland. Full names for the variables are given in ‘Materials and methods’.

Redundancy analysis for the covariation between leaf nutrients and environmental factors showed that 62.1% of total variation in leaf nutrients was explained by climate and soil variables (Fig. 4). Leaf N and P were mainly explained by MAT, soil pH, soil available P and soil C:N ratio, while leaf N:P ratio was related to TWQ (mean temperature of warmest quarter), soil organic C, soil N and soil available N (Fig. 4). Leaf N, P and N:P ratio of *P. australis* in different climatic regions were explained by different environmental factors (Fig. 4).

### Relationships between leaf N, P and N:P ratio and environmental variables in different climatic regions

Leaf N of *P. australis* in different climatic regions showed different geographic patterns, i.e. it decreased with latitude in temperate region (*t* = −2.36, *p* = 0.018) and increased with altitude in subtropical region (*t* = 2.21, *p* = 0.027). Leaf P and N:P ratio did not have any clear geographic patterns in all the three climatic regions (Fig. 2).

Leaf N, P and N:P ratio of *P. australis* in the three climatic regions were predicted by few climate or soil factors (Fig. 3). Leaf N in temperate region (*t* = 1.99, *p* = 0.046) and leaf P in subtropical region (*t* = 2.29, *p* = 0.022) increased with increasing soil available P. Leaf N:P ratio in subtropical region decreased with soil pH (*t* = −3.02, *p* = 0.003).

## Discussion

Leaf N and N:P ratio of *P. australis* were highest in highlands, which may be due to the much lower temperature and higher soil N in this climatic region (Supplementary Fig. S1). Low temperature tended to aid the physiological acclimation of N and P, which was predicted by the Temperature-Dependent Physiology Hypothesis^11^,^15^,^30^. The relatively low leaf P of *P. australis* in highlands was probably related to P limitation, because the mean of leaf N:P ratio (18.1) of *P. australis* was above 16 (N:P ratio < 14 indicates N limitation, while N:P ratio > 16 indicates P limitation)^4^,^5^, and soil P was low in this region (Supplementary Fig. S1).

Leaf N and P of *P. australis* found in this study seemed to be higher than that of this species in European wetlands (mean: N ≈ 14.0 mg g^−1^; P ≈ 1.0 mg g^−1^), and leaf N:P ratio was similar in these two areas^31^ (mean ≈ 15.0). The difference in leaf N and P was probably because of the differences in environmental variables (both climate and soil) between wetlands in China and Europe. Meanwhile, *P. australis*, similar to other aquatic macrophytes (*t*-tests, *p* = 0.477), had a higher leaf N than terrestrial plant species (Supplementary Table S2; *t*-tests, *p* < 0.001). Compared with other aquatic macrophytes, *P. australis* had lower leaf P (*t*-tests, *p* < 0.001) and higher leaf N:P ratio (*t*-tests, *p* < 0.001), which was more similar to terrestrial plants (Supplementary Table S2). Since leaf N:P ratio has frequently been found to be negatively related to relative growth rate of plants^1^,^2^, it implies that *P. australis* had a lower relative growth rate which is similar to terrestrial plants rather than to most of aquatic macrophytes.

Leaf N, P and N:P ratio of terrestrial plants have been extensively investigated^11^,^12^,^14^,^23^,^24^,^32^,^33^. However, the variation in stoichiometry of wetland plants, especially for intraspecific variation, was unclear. In this study, we investigated the variation in leaf nutrients of a single wetland species across large geographic scales in relation to climate and soil factors. Inconsistent with our expectation, weak geographic patterns in leaf nutrients of *P. australis*, except for leaf N:P ratio along altitude, were found across the species range in China (Fig. 2). Negative relationship between leaf N and MAT was consistent with what has been largely found among species in terrestrial ecosystems^11^,^12^,^14^,^33^, which supported the Temperature-Dependent Physiology Hypothesis^11^,^15^. Moreover, strong relationships between leaf N, P and soil properties (soil pH, soil C:N ratio and soil available P) were consistent with the Environmental Nutrient Supply Hypothesis^17^,^18^,^19^. These facts emphasized the importance of temperature and soil factors, most of which are correlated with each other (Supplementary Table S3), in determining leaf nutrients of *P. australis*. The influences of soil and climate on leaf nutrients had shown weak geographic patterns in leaf stoichiometry of *P. australis*, which might be related to the following two main reasons. First, as azonal vegetation, wetland plants were relatively weakly influenced by climate factors compared with local abiotic or biotic factors^20^,^27^. It explained why there were weak linkages between leaf P and MAT or latitude (Figs 2 and 3). Second, although leaf N and P were strongly correlated with soil properties, such as soil pH, soil C:N ratio and soil available P, there was no clear geographic gradients in nutrient limitation in wetlands.

The weak geographic patterns in leaf nutrients of *P. australis* are different from what has been previously found in terrestrial plants across China^12^,^33^ and the globe^11^,^14^, in which leaf N and P increase with latitude and altitude. The comparison of other wetland plants to terrestrial plants also supported this, although there are some differences in the variation in leaf nutrients among different species (Supplementary Table S4). It may suggest that implicit mechanism of variation in stoichiometry of wetland plants might differ from terrestrial plants. Since wetland plants were relatively little investigated, there are great needs to explore geographic variation in leaf nutrients of wetland plants^34^. Moreover, our study found the greater effects of soil on stoichiometry of *P. australis* than climate, which showed some lights on the causes of intraspecific variation in leaf stoichiometry of widely distributed species^21^,^35^. Furthermore, *P. australis* is a dominant species in many wetland ecosystems^27^, and leaf chemistry is strongly linked to nutrient cycling^2^, therefore, this study will help us to understand the effects of environment change on wetland ecosystem functioning.

There was a negative latitudinal trend in leaf N in temperate region and positive altitudinal pattern in leaf N in subtropical region. Leaf N of *P. australis* in temperate region and leaf P in subtropical region increased with increasing soil available P. However, leaf N:P ratio in subtropical region decreased with soil pH. In other words, relationships between leaf N, P and N:P ratio and environmental variables differed among climatic regions. This was probably not due to the limited ranges of leaf N, P and N:P ratio in some climatic regions in this study. Because sampling sites were almost evenly distributed across each region (Supplementary Table S5), and there were no obvious differences in the ranges of leaf N, P and N:P ratio among the three climatic regions (Fig. 1). In different climatic regions, multiple environmental variables, including soil nutrients and climate factors, are likely to shape leaf stoichiometry variation. This is consistent with the study of Kang *et al*.^35^, which showed significant geographic patterns of leaf N and P in temperate region instead of in Mediterranean and boreal regions. Therefore, we conclude that climatic regions modulated the responses of leaf N, P and N:P ratio to environmental gradients.

Plant leaf N, P and N:P ratio varied among climatic regions, functional groups and ecosystems^12^,^13^. Previous studies compared the variation in leaf N, P and N:P ratio along environmental gradients among taxa, functional groups, life forms and ecosystems^11^,^12^,^14^,^24^,^34^. In this study, we investigated the geographic variation in leaf N, P and N:P ratio in different climatic regions, and found that leaf nutrients in different climatic regions responded differently to environmental variables. Different climatic regions, similar to different sampling zone within species ranges, resulted in different patterns of variation in leaf nutrient along environmental gradients^25^. This provides insight into the mechanism of variation in plant leaf nutrients. In addition, our results suggest that it is necessary to consider the effects of climatic regions when studying the relationships between leaf nutrients and the environment, which might help us better understand the responses of plant nutrients to environmental changes.

In this study, we investigated the intraspecific variation in leaf N and P along wide environmental gradients with the cosmopolitan wetland plant *P. australis*. We found no clear geographic patterns in leaf nutrients of *P. australis*, except for leaf N:P ratio along altitude, across the species range in China. This differs from what has been previously reported in terrestrial plants, for which leaf N and P increase with latitude and altitude. Moreover, leaf N and N:P ratio decreased with increasing MAT, while leaf N and P were strongly correlated with soil pH, soil C:N ratio and soil available P. This did not explain the geographic trends in leaf nutrients of *P. australis*, but suggests that climate and soil variables are able to shape the intraspecific patterns of leaf N and P stoichiometry. Furthermore, we found that the relationships between leaf N, P and N:P ratio and environmental variables differed among climatic regions, indicating that leaf nutrients in different climatic regions responded differently to environmental variables. Our findings have important implications for understanding the determinants of variation in plant tissue nutrients and predicting plant responses to environmental changes. Since we investigated the influences of climate and soil on only one widespread wetland plant, more studies on other wetlands species are needed to gain insight into the effects of climate change and N deposition on wetland ecosystem functioning.

## Materials and Methods

### Data set of leaf N and P in *P. australis*

Data for leaf N and P of *P. australis* and climatic and soil variables across China were obtained from published literature and our field investigation. We collected the data from peer-reviewed papers, dissertations and books both in Chinese and English, and removed the duplicates among them. To minimize errors caused by spatial and temporal variability, we collected data based on the following criteria: 1) natural population of *P. australis*, excluding those from greenhouse and field experiments; 2) wetland habitats, excluding terrestrial habitats; 3) data were collected in growing seasons (July to September); 4) data were obtained only for sites without obvious disturbances. For some studies having several measures from July to September, the averages were calculated. Data from our field investigation also met these criteria. Mature leaves of *P. australis* were collected from 16 sites in natural wetlands across China during the growing seasons. The detailed sampling methods for our field investigation were described in Hu *et al*.^29^. The final data set included 58 sampling sites from 29 publications and our field investigation (Supplementary Table S5; Data S1). These sampling sites spanned latitudes from 22.1°N to 47.6°N and longitudes from 83.5°E to 133.5°E, covering most of the geographic ranges of *P. australis* in China (Supplementary Table S5). They covered large climatic gradients with MAT −0.4 to 22.7 °C and MAP 40 to 2223 mm.

Sampling sites were distributed in three climatic regions—subtropical, temperate and highland; highlands represented the climate of the Tibetan Plateau^36^. The three climatic regions differed in both climate and soil variables (Supplementary Fig. S1). Given the large variability in traits of *P. australis* at small scales^29^, leaf N and P were collected and measured at plot level, i.e. 1–4 plots within each sampling site. We identified the plot trait data according to the information of published studies. For some studies which were conducted at the same sites but by different authors and in different years, we treated data from these studies as independent plots. In total, we had 100 plots, i.e. 100 records for leaf N, P and N:P ratio.

### Leaf N and P

We collected leaf N and P of *P. australis* from published papers based on the same criteria as mentioned above. In our field investigation, leaf N was determined with an elemental analyzer (vario PYRO cube; Elemental, Germany), while leaf P was determined using ascorbic acid colorimetric method after H_2_SO_4_ digestion as described by Bao (2005)^37^. Leaf N and P were all expressed on a mass basis (mg g^−1^). Leaf N:P ratio was also collected from publications or calculated by the ratio of leaf N to P.

### Environmental variables

Latitude, longitude and altitude of sampling sites were recorded in our investigation or collected from publications. MAT and MAP were obtained directly from literature, and if they were not accessible in literature, we obtained them from the WorldClim database (http://www.worldclim.org/) according to geographical coordinate of each site^38^. Notably, for most of sampling sites in highlands (the Tibetan Plateau), MAT rather than latitude and longitude were provided, in which case MAT was the only climate variable (Supplementary Table S5). Temperature seasonality (TS), precipitation seasonality (PS), mean diurnal range (DRT, °C), mean temperature of warmest quarter (TWQ, °C), and mean precipitation of warmest quarter (PWQ, mm) for each site were also obtained from the WorldClim. We collected soil variables, including soil pH, soil electrical conductivity (soil EC, ms cm^−1^), soil N (mg g^−1^), soil P (mg g^−1^), soil C:N ratio, soil organic C (mg g^−1^), soil available N (mg kg^−1^) and soil available P (mg kg^−1^), of each plot from the literature. In our field investigation, we determined these eight soil variables using the methods described in Hu *et al*.^29^.

### Statistical analyses

We first calculated the mean, standard deviation (SD) and coefficient of variation (CV) of leaf N and P in *P. australis* overall and in three climatic regions. Before the following statistical analyses, MAP and PWQ were square root-transformed, while leaf P, altitude, soil N, soil P, soil C:N ratio, soil EC, soil organic C, soil available N, soil available P, DRT, PS were log_10_-transformed. Leaf N, leaf N:P ratio, latitude, MAT, TS, TWQ and soil pH were not transformed as they showed approximately normal distributions.

We carried out one-way ANOVAs and post hoc tests (Tukey’s HSD) to address the differences in leaf N, P and N:P ratio among the three climatic regions. Because our data has a multilevel structure (i.e. variance at the plot level is nested within that at the site level), we used multilevel models to address the effects of environmental variables on leaf N, P and N:P ratio. Each climate or soil variable was used as a predictor and fixed factor, and site as a random factor. We conducted the multilevel models with *lmer* function in lme4 package of R, and parameter estimates were based on restricted maximum likelihood (REML)^39^. Finally, to explore the effects of soil and climate on the matrix of leaf N, P and N:P ratio, we performed redundancy analysis with *rda* function in R package vegan^40^. All analyses were performed in R 3.2.3 (R Core Team 2015)^41^.

## Additional Information

**How to cite this article:** Hu, Y.-K. *et al*. Intraspecific N and P stoichiometry of *Phragmites australis*: geographic patterns and variation among climatic regions. *Sci. Rep.* **7**, 43018; doi: 10.1038/srep43018 (2017).

**Publisher's note:** Springer Nature remains neutral with regard to jurisdictional claims in published maps and institutional affiliations.

## Supplementary Material

Supplementary Information
