# Supplementary material for: Intraspecific N and P stoichiometry of Phragmites australis: geographic patterns and variation among climatic regions
Source: Sci Rep. 2017 Feb 24;7:43018. doi: 10.1038/srep43018 (PMC5324045; doi:10.1038/srep43018)
Supplement: Supplementary Information [file srep43018-s1.doc]

**Intraspecific N and P stoichiometry of *Phragmites australis*: geographic patterns and variation among climatic regions**

**Yu-Kun Hu, Ya-Lin Zhang, Guo-Fang Liu, Xu Pan, Xuejun Yang, Wen-Bing Li, Wen-Hong Dai, Shuang-Li Tang, Tao Xiao, Ling-Yun Chen, Wei Xiong, Yao-Bin Song, Ming Dong**

**Supplementary information**

**Supplementary Table S1. Relationships between environmental variables and leaf nutrients of *Phragmites australis*. Variance explained (R^2^) by each fixed factor was calculated in linear mixed models (Nakagawa & Schielzeth 2013).**

| Environmental variables | R^2^ | | |
| --- | --- | --- | --- |
|  | Leaf N | Leaf P | Leaf N:P |
| Latitude | 0.021 | 0.064 | 0.034 |
| Altitude | 0.034 | 0.002 | **0.127** |
| MAT | **0.082** | 0.003 | **0.096** |
| MAP | 0.009 | **0.078** | 0.060 |
| TS | 0.032 | 0.053 | 0.018 |
| PS | 0.069 | 0.018 | 0.019 |
| DRT | <0.001 | 0.044 | **0.119** |
| TWQ | 0.002 | 0.033 | **0.094** |
| PWQ | 0.032 | **0.150** | 0.088 |
| Soil pH | **0.127** | 0.073 | 0.002 |
| Soil EC | 0.076 | 0.039 | 0.051 |
| Soil N | 0.007 | 0.007 | 0.031 |
| Soil P | 0.030 | 0.015 | 0.002 |
| Soil C:N | **0.123** | **0.169** | 0.016 |
| Soil OC | <0.001 | 0.057 | 0.073 |
| Soil AN | 0.009 | 0.029 | 0.076 |
| Soil AP | **0.102** | **0.088** | 0.003 |

Values of R^2^ were calculated with *r.squaredGLMM* function in R package ‘MuMIn’ (Bartoń 2016). Values in bold indicate significance at *p* < 0.05. MAT, mean annual temperature; MAP, mean annual precipitation; DRT, mean diurnal range; TS, temperature seasonality; TWQ, mean temperature of warmest quarter; PS, precipitation seasonality; PWQ, mean precipitation of warmest quarter; soil EC, soil electrical conductivity; soil OC, soil organic carbon; soil AN, soil available N; soil AP, soil available P.

**Supplementary Table S2. Comparison of leaf N, P and N:P ratio in different studies.**

| Study | Leaf N (mg g^-1^) | | | Leaf P (mg g^-1^) | | | Leaf N:P | | |
| --- | --- | --- | --- | --- | --- | --- | --- | --- | --- |
|  | Mean | SD | *N* | Mean | SD | *N* | Mean | SD | *N* |
| This study | 26.6 | 8.6 | 92 | 1.8 | 0.8 | 86 | 16.2 | 4.3 | 80 |
| Xia et al. (2014) | 25.9 | 10.4 | 681 | 3.3* | 1.6 | 681 | 9.5* | 6.0 | 681 |
| Han et al. (2005) | 20.2* | 8.4 | 554 | 1.5* | 1.0 | 745 | 16.3 | 9.3 | 547 |
| Reich and Oleksyn (2004) | 20.1* | 8.7 | 1251 | 1.8 | 1.1 | 923 | 13.8* | 9.5 | 894 |

SD, standard deviation. Significant differences between this study and the others were marked with * (*p* < 0.05). Information for the studies: this study, *P*. *australis* across China; Xia *et al*. (2004), 122 aquatic macrophytes across China; Han *et al*. (2005), 753 terrestrial plant species across China; Reich & Oleksyn (2004), 1280 terrestrial plant species across the globe.

**Supplementary Table S3. Results for Pearson’s correlation analysis among environmental variables at plot level.**

|  | Lat | Alt | MAT | MAP | DRT | TS | TWQ | PS | PWQ | Soil pH | Soil EC | Soil N | Soil P | Soil C:N | Soil OC | Soil AN | Soil AP |
| --- | --- | --- | --- | --- | --- | --- | --- | --- | --- | --- | --- | --- | --- | --- | --- | --- | --- |
| Lat |  | 80 | 80 | 80 | 80 | 80 | 80 | 80 | 80 | 40 | 40 | 49 | 48 | 40 | 40 | 40 | 40 |
| Alt | **0.26** |  | 80 | 80 | 80 | 80 | 80 | 80 | 80 | 40 | 40 | 49 | 48 | 40 | 40 | 40 | 40 |
| MAT | **-0.84** | **-0.56** |  | 80 | 80 | 80 | 80 | 80 | 80 | 40 | 40 | 49 | 48 | 40 | 40 | 40 | 40 |
| MAP | **-0.82** | **-0.50** | **0.79** |  | 80 | 80 | 80 | 80 | 80 | 40 | 40 | 49 | 48 | 40 | 40 | 40 | 40 |
| DRT | **0.72** | **0.68** | **-0.80** | **-0.88** |  | 80 | 80 | 80 | 80 | 40 | 40 | 49 | 48 | 40 | 40 | 40 | 40 |
| TS | **0.97** | 0.15 | **-0.74** | **-0.74** | **0.62** |  | 80 | 80 | 80 | 40 | 40 | 49 | 48 | 40 | 40 | 40 | 40 |
| TWQ | **-0.40** | **-0.70** | **0.81** | **0.50** | **-0.65** | **-0.23** |  | 80 | 80 | 40 | 40 | 49 | 48 | 40 | 40 | 40 | 40 |
| PS | **0.42** | **0.34** | **-0.47** | **-0.37** | **0.57** | **0.28** | **-0.46** |  | 80 | 40 | 40 | 49 | 48 | 40 | 40 | 40 | 40 |
| PWQ | **-0.70** | **-0.46** | **0.69** | **0.93** | **-0.75** | **-0.67** | **0.41** | -0.06 |  | 40 | 40 | 49 | 48 | 40 | 40 | 40 | 40 |
| Soil pH | **0.66** | 0.23 | **-0.51** | **-0.71** | **0.68** | **0.57** | -0.13 | **0.37** | **-0.56** |  | 40 | 40 | 40 | 40 | 40 | 40 | 40 |
| Soil EC | **0.62** | **0.36** | **-0.65** | **-0.87** | **0.73** | **0.57** | -0.25 | 0.02 | **-0.85** | **0.57** |  | 40 | 40 | 40 | 40 | 40 | 40 |
| Soil N | 0.07 | **0.28** | **-0.39** | -0.10 | **0.29** | 0.01 | **-0.51** | 0.06 | -0.10 | -0.05 | -0.03 |  | 48 | 40 | 40 | 40 | 40 |
| Soil P | 0.06 | **0.34** | -0.22 | -0.21 | 0.24 | 0.04 | -0.25 | -0.04 | -0.18 | **0.39** | 0.18 | **0.32** |  | 40 | 40 | 40 | 40 |
| Soil C:N | **0.66** | 0.14 | **-0.48** | **-0.72** | **0.68** | **0.62** | -0.03 | **0.31** | **-0.61** | **0.68** | **0.69** | -0.30 | 0.07 |  | 40 | 40 | 40 |
| Soil OC | 0.13 | 0.29 | **-0.49** | -0.21 | **0.34** | 0.03 | **-0.57** | 0.08 | -0.19 | 0.04 | 0.07 | **0.97** | 0.27 | -0.16 |  | 40 | 40 |
| Soil AN | -0.05 | 0.17 | **-0.31** | -0.03 | 0.14 | -0.11 | **-0.47** | -0.05 | -0.06 | -0.20 | -0.09 | **0.97** | 0.20 | **-0.39** | **0.95** |  | 40 |
| Soil AP | -0.20 | 0.24 | -0.15 | -0.01 | <0.01 | -0.23 | **-0.35** | -0.13 | -0.02 | 0.03 | 0.18 | **0.37** | **0.58** | -0.23 | **0.37** | **0.36** |  |

Pearson’s correlation coefficients are given in the lower left, and values in bold indicate significance at *p* < 0.05. Sample size for each correlation is given in the upper right. Lat, MAT, TS, TWQ and soil pH were not transformed; MAP and PWQ were square root-transformed; other variables were log_10_-transformed. Lat, latitude; Alt, altitude. The full names of other variables were given in Supplementary Table S2.

**Supplementary Table S4. Overview of previous studies investigating intraspecific variation in leaf N, P and N:P ratio. The selection of studies is not exhaustive.**

| **Species** | **Habitat** | **Functional type** | **Study region** | **N mean (mg g^-1^)** | **N range (mg g^-1^)** | **P mean (mg g^-1^)** | **P range (mg g^-1^)** | **N:P mean** | **N:P range** | **The linkages to geographical, climate and soil factors** | **Study** |
| --- | --- | --- | --- | --- | --- | --- | --- | --- | --- | --- | --- |
| *Oryza ruﬁpogon* | Wetland | Grass | Southern China | 12.5 | 6.5-20.0 | 1.2 | 0.7-1.9 | 9.5 | 6.0-15.2 | Leaf P decreased with latitude, and increased with MAT and soil P. Leaf N:P increased with latitude and decreased with MAT. | Zhou et al. 2014 |
| *Spartina alterniflora* | Wetland | Grass | The coast of Louisiana, USA | 14.2 | - | 1.3 | - | 25.71 | - | Negative relationships between leaf P and water pH, and positive between leaf N and water ammonia-N | Tobias et al. 2014 |
| *Spartina patens* | Wetland | Grass | The coast of Louisiana, USA | 10.7 | - | 0.9 | - | 25.58 | - | - | Tobias et al. 2014 |
| *Ranunculus natans* | Wetland | Herb | Northwest China | 32.3 | 20.1-55.4 | - | - | - | - | Weak relationships between leaf N and MAT or MAP, but leaf N showed negative relationships with temperature of water body. | Li et al. 2015 |
| *Sarracenia purpurea* | Wetland | Herb | Massachusetts and Vermont, USA | ? | ? | ? | ? | ? | ? | Leaf N:P increased with elevation | Gotelli et al. 2008 |
| *Sphagnum* spp. | Wetland | Herb | Massachusetts and Vermont, USA | ? | ? | ? | ? | ? | ? | Leaf N:P increased with elevation | Gotelli et al. 2008 |
| *Chamaedaphne calyculata* | Wetland | Shrub | Massachusetts and Vermont, USA | ? | ? | ? | ? | ? | ? | No geographical patterns | Gotelli et al. 2008 |
| *Rhizophora* spp. | Wetland | Tree | Northern Australian | 9.4 | 8.1-10.8 | 0.85 | 0.76-1.07 | - | - | - | Boto & Wellington 1983 |
| *Artemisia mongolica* | Terrestrial | Herb | Northern China | 16.7 | 8.47-34.94 | 1.9 | 0.32-4.63 | 11.87 | 2.68-36.71 | - | Yang et al. 2015 |
| *Artemisia rubripes* | Terrestrial | Herb | Northern China | 13.86 | 7.57-25.03 | 1.42 | 0.42-3.93 | 12.8 | 3.26-33.84 | - | Yang et al. 2015 |
| *Artemisia sacrorum* | Terrestrial | Herb | Northern China | 14.59 | 8.29-23.31 | 1.01 | 0.33-2.55 | 16.75 | 4.83-39.67 | - | Yang et al. 2015 |
| *Artemisia scoparia* | Terrestrial | Herb | Northern China | 13.99 | 6.26-26.89 | 1.32 | 0.32-3.11 | 13.25 | 3.45-41.26 | - | Yang et al. 2015 |
| *Trifolium alpinum* | Terrestrial | Herb | Central French Alps | 32.5 | 26.0-40.5 | - | - | - | - | Leaf N decreases with temperature | Albert et al. 2010 |
| *Hippophae rhamnoides* | Terrestrial | Shrub | Northern China | 36.57 | 32.60-43.61 | 2.08 | 1.50-2.69 | 17.81 | 13.46-23.07 | Relationships between leaf N, N:P and latitude followed convex curves. Leaf P was positive related to MAP | Li et al. 2014 |
| *Reaumuria soongorica* | Terrestrial | Shrub | Northwest China | 10.6 | 2.09-21.4 | 0.73 | 0.16-2.56 | 15.7 | 6.60-27.00 | Relationships between leaf N, P and latitude followed convex curves. | He et al. 2015 |
| *Metrosideros polymorpha* | Terrestrial | Tree | Hawaii, USA | ? | ? | - | - | - | - | Leaf N increases with elevation | Cordell et al. 1998 |
| *Picea abies* | Terrestrial | Tree | Europe | 13.37 | 9.45-19.15 | 1.41 | 0.40-3.50 | 9.76 | 4.99-19.29 | Leaf N:P increases with MAT; leaf N and P show different relationships with climate in different regions | Kang et al. 2011 |
| *Pinus sylvestris* | Terrestrial | Tree | Europe | ? | ? | ? | ? | ? | ? | Leaf N decreases with latitude | Oleksyn et al. 2003 |
| *Pinus sylvestris* | Terrestrial | Tree | Europe | ? | ? | ? | ? | 10.08 | ? | Leaf N and P were higher in central latitudes, while leaf N:P decreased with latitude. Leaf N and N:P were positively correlated with atmospheric N deposition. | Sardans et al. 2016 |
| *Quercus* spp. | Terrestrial | Tree | China | 17.27 | 7.81-32.26 | 1.54 | 0.40-3.01 | 13.96 | 6.07-34.08 | leaf N and P increase with latitude; leaf P decrease with MAT; | Wu et al. 2012 |
| *Quercus acutissima* | Terrestrial | Tree | China | 16.23 | - | 1.41 | - | 14 | - | - | Wu et al. 2012 |
| *Quercus aliena* var. *acutiserrata* | Terrestrial | Tree | China | 20.29 | - | 1.53 | - | 13.34 | - | - | Wu et al. 2012 |
| *Quercus mongolica* | Terrestrial | Tree | China | 21.03 | - | 2.08 | - | 11.63 | - | - | Wu et al. 2012 |
| *Quercus variabilis* | Terrestrial | Tree | China | 18.33 | - | 1.18 | - | 16.56 | - | - | Wu et al. 2012 |
| *Quercus variabilis* | Terrestrial | Tree | China | 19 | 13.17-24.56 | 1.03 | 0.47-2.25 | 20.48 | 8.91-37.61 | Leaf N and P decreased with MAT and increased with aridity. Positive latitudinal trends in leaf N and P. | Sun et al. 2015 |
| *Quercus wutaishanica* | Terrestrial | Tree | China | 23.52 | - | 1.74 | - | 14.57 | - | - | Wu et al. 2012 |
| *Quercus wutaishanica* | Terrestrial | Tree | The Loess Plateau, Northern China | 23.59 | 17.60-33.70 | - | - | 21.78 | 13.15-41.76 | Leaf N was correlated with MAP and soil P and K. | Xing et al. 2016 |
| *Quercus ilex* | Terrestrial | Tree | Europe - Mediterranean | 13.63 | 11.0-18.0 | - | - | - | - | Leaf N shows weak relationships with climate factors. | Niinemets et al. 2015 |

Symbol ‘-’ means trait values or relationships not investigated, while ‘?’ means investigated but not given directly in these studies.

**Supplementary Table S5. Location and climate information of sampling sites for *Phragmites australis*.**

| Site | Latitude (°N) | Longitude  (°E) | Altitude (m) | MAT (°C) | MAP (mm) | Region | Source |
| --- | --- | --- | --- | --- | --- | --- | --- |
| 1 | 22.2 | 113.6 | 21 | 22.7 | 1824 | Subtropical | Literature |
| 2 | 22.3 | 114.2 | 174 | 21.9 | 2223 | Subtropical | Literature |
| 3 | 24.9 | 102.7 | 1898 | 15.7 | 1000 | Subtropical | Investigation |
| 4 | 26.0 | 119.6 | 6 | 19.6 | 1348 | Subtropical | Literature |
| 5 | 26.6 | 118.9 | 398 | 17.4 | 1702 | Subtropical | Investigation |
| 6 | 29.4 | 113.0 | 25 | 16.7 | 1350 | Subtropical | Investigation |
| 7 | 29.8 | 113.4 | 23 | 17.0 | 1265 | Subtropical | Literature |
| 8 | 29.9 | 114.0 | 44 | 17.1 | 1347 | Subtropical | Literature |
| 9 | 30.3 | 120.1 | 9 | 16.8 | 1370 | Subtropical | Literature |
| 10 | 30.3 | 121.1 | 6 | 16.6 | 1234 | Subtropical | Literature |
| 11 | 30.4 | 114.2 | 25 | 17.2 | 1268 | Subtropical | Literature |
| 12 | 30.5 | 114.4 | 25 | 17.2 | 1262 | Subtropical | Literature |
| 13 | 30.5 | 113.0 | 31 | 16.6 | 1095 | Subtropical | Literature |
| 14 | 30.5 | 114.2 | 24 | 17.1 | 1241 | Subtropical | Literature |
| 15 | 31.2 | 122.0 | 8 | 16.6 | 1231 | Subtropical | Literature |
| 16 | 31.4 | 119.4 | 39 | 15.7 | 1152 | Subtropical | Literature |
| 17 | 31.5 | 121.8 | 2 | 15.8 | 1037 | Subtropical | Literature |
| 18 | 31.7 | 117.4 | 8 | 16.2 | 955 | Subtropical | Investigation |
| 19 | 33.0 | 111.5 | 148 | 15.1 | 788 | Subtropical | Investigation |
| 20 | 33.1 | 107.1 | 483 | 14.8 | 829 | Subtropical | Investigation |
| 21 | 34.1 | 118.2 | 21 | 14.7 | 732 | Temperate | Literature |
| 22 | 35.1 | 116.7 | 54 | 13.7 | 684 | Temperate | Investigation |
| 23 | 36.3 | 120.1 | 2 | 12.5 | 710 | Temperate | Literature |
| 24 | 37.6 | 115.6 | 22 | 12.7 | 519 | Temperate | Investigation |
| 25 | 38.4 | 90.2 | 3101 | 0.9 | 55 | Temperate | Literature |
| 26 | 38.9 | 115.9 | 7 | 12.8 | 499 | Temperate | Literature |
| 27 | 39.0 | 108.2 | 1423 | 7.0 | 285 | Temperate | Literature |
| 28 | 40.3 | 93.8 | 1006 | 9.9 | 40 | Temperate | Investigation |
| 29 | 40.4 | 115.8 | 471 | 11.8 | 625 | Temperate | Investigation |
| 30 | 40.8 | 108.7 | 1017 | 6.7 | 224 | Temperate | Investigation |
| 31 | 41.1 | 122.1 | 3 | 9.1 | 631 | Temperate | Literature |
| 32 | 43.3 | 116.9 | 1241 | 2.0 | 375 | Temperate | Investigation |
| 33 | 43.4 | 116.4 | 1190 | -0.4 | 350 | Temperate | Literature |
| 34 | 44.0 | 122.3 | 169 | 6.1 | 395 | Temperate | Literature |
| 35 | 44.7 | 83.4 | 229 | 7.8 | 91 | Temperate | Investigation |
| 36 | 46.3 | 123.0 | 139 | 4.0 | 400 | Temperate | Investigation |
| 37 | 47.6 | 133.5 | 54 | 2.2 | 616 | Temperate | Literature |
| 38 | 33.9 | 102.8 | 3435 | 1.6 | 648 | Highland | Investigation |
| 39 | 37.2 | 96.9 | 2825 | 3.9 | 209 | Highland | Investigation |
| 40 |  |  |  | 1.9 |  | Highland | Literature |
| 41 |  |  |  | 4.2 |  | Highland | Literature |
| 42 |  |  |  | 0.9 |  | Highland | Literature |
| 43 |  |  |  | 6.0 |  | Highland | Literature |
| 44 |  |  |  | 5.5 |  | Highland | Literature |
| 45 |  |  |  | 3.6 |  | Highland | Literature |
| 46 |  |  |  | 4.2 |  | Highland | Literature |
| 47 |  |  |  | 6.0 |  | Highland | Literature |
| 48 |  |  |  | 5.1 |  | Highland | Literature |
| 49 |  |  |  | 10.0 |  | Highland | Literature |
| 50 |  |  |  | 4.6 |  | Highland | Literature |
| 51 |  |  |  | 2.7 |  | Highland | Literature |
| 52 |  |  |  | 3.0 |  | Highland | Literature |
| 53 |  |  |  | 3.0 |  | Highland | Literature |
| 54 |  |  |  | 1.0 |  | Highland | Literature |
| 55 |  |  |  | 1.3 |  | Highland | Literature |
| 56 |  |  |  | 3.5 |  | Highland | Literature |
| 57 |  |  |  | 1.0 |  | Highland | Literature |
| 58 |  |  |  | 4.4 |  | Highland | Literature |

Notably, 19 sampling sites (site 40-58) in highlands (the Tibetan Plateau) from publication data were lack of geographic information. MAT, mean annual temperature; MAP, mean annual precipitation.

**
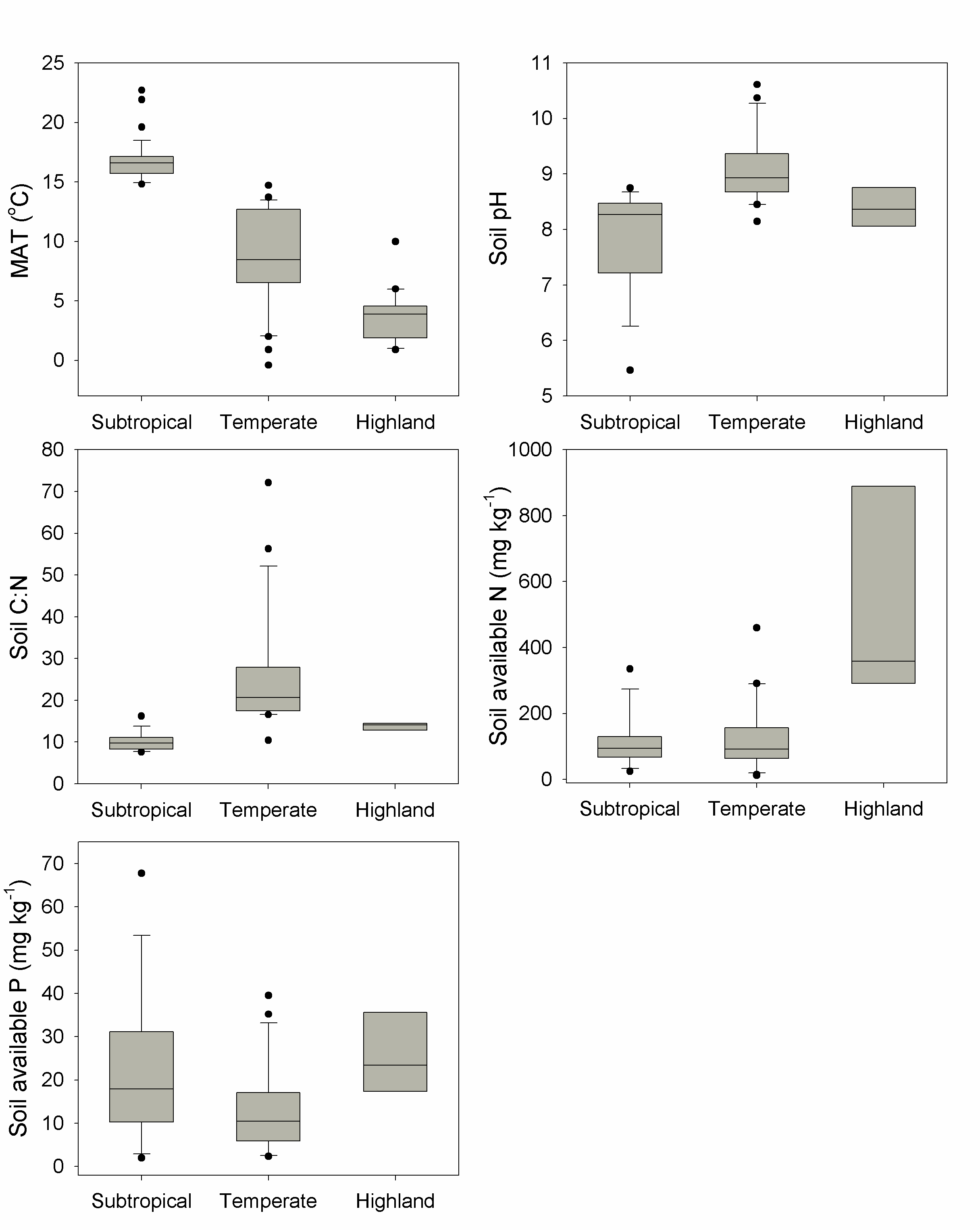
**

**Supplementary Figure S1.** Climate and soil variables at the three climatic regions. MAT, mean annual temperature. Notably, except MAT, data for environmental variables in highlands are few.

**References**

Albert, C. H. *et al.* Intraspecific functional variability: extent, structure and sources of variation. *J. Ecol.* 98, 604-613 (2010).

Bartoń, K. MuMIn: Multi-Model Inference. R package version 1.15.6 (2016).

Boto, K. G. & Wellington, J. T. Phosphorus and nitrogen nutritional status of a northern Australian mangrove forest. *Mar. Ecol-Prog. Ser.* 11, 63-69 (1983).

Cordell, S., Goldstein, G., Mueller-Dombois, D., Webb, D. & Vitousek, P. M. Physiological and morphological variation in *Metrosideros polymorpha*, a dominant Hawaiian tree species, along an altitudinal gradient: the role of phenotypic plasticity. *Oecologia* 113, 188-196 (1998).

Gotelli, N. J. *et al.* Geographic variation in nutrient availability, stoichiometry, and metal concentrations of plants and pore-water in ombrotrophic bogs in New England, USA. *Wetlands* 28, 827-840 (2008).

Han, W. X., Fang, J. Y., Guo, D. L. & Zhang Y. Leaf nitrogen and phosphorus stoichiometry across 753 terrestrial plant species in China. *New Phytol.* **168**, 377-385 (2005).

He, M. *et al.* Nutrient levels within leaves, stems, and roots of the xeric species *Reaumuria soongorica* in relation to geographical, climatic, and soil conditions. Ecol. Evol. 5, 1494-1503 (2015).

Kang, H. *et al.* Variation in leaf nitrogen and phosphorus stoichiometry in *Picea abies* across Europe: An analysis based on local observations. *Forest Ecol. Manage.* 261, 195-202 (2011).

Li, X., Sun, K. & Li, F. Y. Variation in leaf nitrogen and phosphorus stoichiometry in the nitrogen-fixing Chinese sea-buckthorn (*Hippophae rhamnoides* L. subsp. *sinensis* Rousi) across northern China. *Ecol. Res.* 29, 723-731 (2014).

Li, Z. *et al.* Spatial patterns of leaf carbon, nitrogen stoichiometry and stable carbon isotope composition of *Ranunculus natans* C.A. Mey. (Ranunculaceae) in the arid zone of northwest China. *Ecol. Eng.* 77, 9-17 (2015).

Nakagawa, S. & Schielzeth, H. A general and simple method for obtaining R^2^ from generalized linear mixed-effects models. *Methods Ecol. Evol.* **4**, 133-142 (2013).

Niinemets, Ü. Is there a species spectrum within the world-wide leaf economics spectrum? Major variations in leaf functional traits in the Mediterranean sclerophyll *Quercus ilex*. *New Phytol.* 205, 79-96 (2015).

Oleksyn, J., Reich, P. B., Zytkowiak, R., Karolewski, P. & Tjoelker, M. G. Nutrient conservation increases with latitude of origin in European *Pinus sylvestris* populations. *Oecologia* 136, 220-235 (2003).

Reich, P. B. & Oleksyn, J. Global patterns of plant leaf N and P in relation to temperature and latitude. *Proc. Nat. Acad. Sci.* **101**, 11001-11006 (2004).

Sardans, J. *et al.* Foliar and soil concentrations and stoichiometry of nitrogen and phosphorous across European *Pinus sylvestris* forests: relationships with climate, N deposition and tree growth. *Funct. Ecol.* 30, 676-689 (2016).

Sun, X., Kang, H., Kattge, J., Gao, Y. & Liu, C. Biogeographic patterns of multi-element stoichiometry of *Quercus variabilis* leaves across China. *Can. J. Forest Res.* 45, 1827-1834 (2015).

Tobias, V. D., Williamson, M. F. & Nyman, J. A. A Comparison of the elemental composition of leaf tissue of *Spartina patens* and *Spartina alternifora* in Louisiana’s coastal marshes. *J. Plant Nutr.* 37, 1327-1344 (2014).

Wu, T., Dong, Y., Yu, M., Geoff Wang, G. & Zeng, D.-H. Leaf nitrogen and phosphorus stoichiometry of *Quercus* species across China. *Forest Ecol. Manage.* 284, 116-123 (2012).

Xia, C. X., Yu, D., Wang, Z. & Xie, D. Stoichiometry patterns of leaf carbon, nitrogen and phosphorous in aquatic macrophytes in eastern China. *Ecol. Eng.* **70**, 406-413 (2014).

Xing, K. *et al.* Determinants of the N content of *Quercus wutaishanica* leaves in the Loess Plateau: a structural equation modeling approach. *Sci. Rep.* 6, 26845; 10.1038/srep26845 (2016).

Yang, X., Huang, Z., Zhang, K., Cornelissen, J. H. C. & Nardoto, G. B. C:N:P stoichiometry of *Artemisia* species and close relatives across northern China: unravelling effects of climate, soil and taxonomy. *J. Ecol.* 103, 1020-1031 (2015).

Zhou, W., Wang, Z., Xing, W. & Liu, G. Plasticity in latitudinal patterns of leaf N and P of *Oryza rufipogon* in China. *Plant Biol.* 16, 917-923 (2014).

**Supplementary Data S1. Literature used for the data set of leaf N and P in *P. australis*.**

Bai, J. H., Wang, Q. G., Gao, H. F., Xiao, R. & Huang, L. B. Dynamic changes of nitrogen contents and nitrogen cycling in *Phragmites australis* of Xianghai marsh wetland. *Wetland Sci.* **8**, 164-168 (In Chinese with English Abstract) (2010).

Cao, Q., Li, M., Yang, H. & Liu, J. J. Dynamic changes of the retention capacity for phosphorus by emergent macrophytes in the Yeyahu wetland. *Acta Sci. Circumst.* **32**, 1874-1881 (In Chinese with English Abstract) (2012).

Duan, X. N. *et al.* Study on the photosynthetic and transpiration properties of wild *Phragmites australis* in the Wuliangsuhai Lake, Inner Mongolia. *Arid Land Geogr.* **27**, 246-251 (In Chinese with English Abstract) (2004).

Hou, X. Y. *Chinese Vegetable Geography and Chemical Elements Analyses of the Dominate Plant Species*. (Science Press, Beijing, 1982).

Huang, L., Wu, Y., Zhang, J., Li, W. & Zhou, J. Z. Distribution of C, N, P and δ^13^C in aquatic plants of some lakes in the middle Yangtze Valley. *Acta Geosci. Sin.* **24**, 515-518 (In Chinese with English Abstract) (2003).

Jia, Q. Y., Zhou, G. S., Zhou, L. & Xie, Y. B. Dynamics of nitrogen content of *Phragmites australis* in Panjin wetland, China. *Chin. J. Plant Ecol.* **32**, 858-864. (In Chinese with English Abstract) (2008)

Lan, Y. *et al.* Litter decomposition of six macrophytes in a eutrophic shallow lake (Baiyangdian Lake, China). *Clean-Soil Air Water*, **40**, 1159-1166 (2012).

Li, L., *et al.* Study of the relationship between specific leaf area and leaf nitrogen content of typical vegetation in Sanjiang wetlands. *J. Harbin Norm. Univ. (Nat. Sci.)*, **26**, 94-97 (In Chinese with English Abstract) (2010).

Lin, X. T., Liang, H. H., Leung, V. & Xu, Z. N. Seasonal variation of nitrogen and phosphorus contents of *Phragmites communis* in Taipa-Coloane wetland in Macao. *Chin. J. Ecol.* **26**, 5-8 (In Chinese with English Abstract) (2007).

Liu, C. Q. *et al.* Dynamics of biomass，nitrogen and phosphorus storage of *Phragmites australis* in Baiyangdian Lake. *Acta Sci. Circumst.* **32**, 1503-1511 (In Chinese with English Abstract) (2012).

Liu, C. E., Yang, Y. X. & Yang, Y. Distribution characteristics and seasonal dynamics of N, P and K in wetland plants in upper shoal of Jiuduansha, Shanghai. *Chin. J. Ecol.* **27**, 1876-1882 (In Chinese with English Abstract) (2008a).

Liu, C. E., Yang, Y. X. & Yang, Y. Distribution, accumulation and development of element P in middle shoal plants of Jiuduansha Wetlands. *Journal of Tongji Univ. (Nat. Sci.)*, **36**, 1537-1541. (In Chinese with English Abstract) (2008b).

Liu, X. X., Li, J. Q. Study on the functional traits of plant leaf in Beijing Yeyahu wetland. *J. Anhui Agricul. Sci.* **36**, 8406-8409 (In Chinese with English Abstract) (2008).

Liu, W. L. Contents of carbon and nitrogen in the wetland ecosystem of the Jiaozhou Bay and their ecological stoichiometry. MS dissertation, Qingdao University. (In Chinese with English Abstract) (2014).

Ren, K. X., Chen, K. N., Huang, W. & Shi, X. Spatial distribution characteristics of soil nitrogen of four types of plant community in Zhucao river mouth wetland into the lake. *J. Lake Sci.* **24**, 849-857 (In Chinese with English Abstract) (2012).

Wang, Z., Xia, C.X., Yu, D. & Wu, Z.G. Low-temperature induced leaf elements accumulation in aquatic macrophytes across Tibetan Plateau. *Ecol. Eng.* **75**, 1-8. (2015).

Wong, P. K., Liang, Y., Liu, N. Y. & Qiu J.W. Palatability of macrophytes to the invasive freshwater snail *Pomacea canaliculata*: differential effects of multiple plant traits. *Freshwater Biol.* **55**, 2023-2031 (2010).

Wu, A. P., Wu, S. K. & Ni, L. Y. Study of macrophytes nitrogen and phosphorus contents of the shallow lakes in the middle reaches of Changjiang River. *Acta Hydrobiol. Sin.* **29**, 406-412 (In Chinese with English Abstract) (2005).

Wu, T. G., Wu, M., Liu, L. & Xiao, J. H. Seasonal variations of leaf nitrogen and phosphorus stoichiometry of three herbaceous species in Hangzhou Bay coastal wetlands, China. *Chin. J. Plant Ecol.* **34**, 23-28 (In Chinese with English Abstract) (2010).

Xie, C. Y., Xu, T. K., Qian, A. A., Ning, J. Y. & Chen, B. The nutrient accumulation and distribution characteristics of the different communities in Xixi wetland ecosystem. *Bull. Sci. Technol.* **28**, 220-227 (In Chinese with English Abstract) (2012).

Yan, Q., He, W. S. & Lu, J. J. Temporal and spatial changes of plant biomass and its N content during vegetation succession at Chongming Dongtan wetland. *Chin. J. Ecol.* **25**, 1019-1023 (In Chinese with English Abstract) (2006).

Yang, Y. X., Liu, C. E., Yang, Y. & Li, K. Distribution characteristics and seasonal dynamics of wetland plant N, P, and K in lower shoal of Jiuduansha at Yangtze River estuary. *Chin. J. Ecol.* **29**, 1277-1288. (In Chinese with English Abstract) (2010).

Yu, H. B. Study on utilization strategy for mineral nutrients of wetland plants on floodplain in the Xilinghe River. MS dissertation, Inner Mongolia University. (In Chinese with English Abstract) (2009).

Zan, X. X. The research of biology and ecological stoichiometry of *Ruditapes philippinarum* and *Phragmites australis* and ecological restoration in Jiaozhou Bay wetland. PhD dissertation, Ocean University of China. (In Chinese with English Abstract) (2013).

Zeng, C. S., Zhang, L. H. & Tong, C. Seasonal dynamics of nitrogen and phosphorus in *Phragmites australis* and *Spartina alterniflora* in the wetlands of Min River Estuary. *Wetland Sci.* **7**, 16-24 (In Chinese with English Abstract) (2009).

Zhao, M. X. *et al.* Ecological stoichiometrical analysis on the strategies of utilization of nitrogen and phosphorus in *Phragmites australis* and *Spartina alterniflora* in Chongming Dongtan wetland. *Guihaia* **32**, 715-722 (In Chinese with English Abstract) (2012).

Zhao, Y., Yang, Z. F., Xia, X. H. & Wang, F. A shallow lake remediation regime with *Phragmites australis*: Incorporating nutrient removal and water evapotranspiration. *Water Res* **46**, 5635-5644 (2012)

Zhang, C. Y., Xu, D. L., Wang, L., Han, B. P. & Yan, S. Relationship between N and P contents in four types of plants and soils in riparian zone of Luomahu Lake. *Res. Soil Water Conserv.* **20**, 38-47. (In Chinese with English Abstract) (2013)

Zhang, Y. M., Yang, Y. F., Wang, L. J. Seasonal dynamics of N element in *Phragmites australis* in the ecosystem of Sanjiang wetland plain. *J. Anhui Agricult. Sci.* **37**, 8148-8150. (In Chinese with English Abstract) (2009).
